# Structural and compositional properties of 2D CH_3_NH_3_PbI_3_ hybrid halide perovskite: a DFT study†

**DOI:** 10.1039/d2ra02874c

**Published:** 2022-09-13

**Authors:** Sandip R. Kumavat, Geeta Sachdeva, Yogesh Sonvane, Sanjeev K. Gupta

**Affiliations:** Advanced Materials Lab, Department of Physics, Sardar Vallabhbhai National Institute of Technology Surat 395007 India yas@phy.svnit.ac.in; Department of Physics, Michigan Technological University Houghton Michigan 49931 USA; Computational Materials and Nanoscience Group, Department of Physics, St. Xavier’s College Ahmedabad 380009 India

## Abstract

Two-dimensional (2D) hybrid halide perovskites have been scrutinized as candidate materials for solar cells because of their tunable structural and compositional properties. Results based on density functional theory demonstrate its thickness-dependent stability. We have observed that the bandgap decreases from the mono- to quad-layer because of the transformation from 2D towards 3D. Due to the transformation, the carrier mobility is lowered with the corresponding smaller effective mass. On the other hand, the multilayer structures have good optical properties with an absorption coefficient of about 10^5^ cm^−1^. The calculated absorption spectra lie between 248 nm and 496 nm, leading to optical activity of the 2D multilayer CH_3_NH_3_PbI_3_ systems in the visible and ultraviolet regions. The strength of the optical absorption increases with an increase in thickness. Overall results from this theoretical study suggest that this 2D multilayer CH_3_NH_3_PbI_3_ is a good candidate for photovoltaic and optoelectronic device applications.

## Abstract 2

We have studied 2D CH_3_NH_3_PbI_3_ and its multilayer halide perovskites. These systems have a high formation energy. The optical properties absorption spectra lie between 248 nm to 496 nm with an absorption coefficient of about 10^5^ cm^−1^.

## Graphical abstract

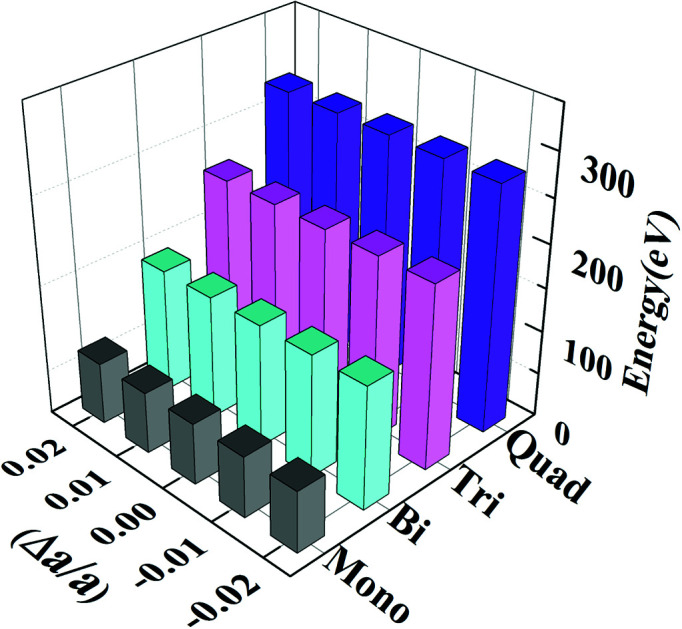

## 1 Introduction

All over the world, population growth and human development has led to industry using much energy, which increases environmental pollution. To overcome this situation many researchers and scientists are working on finding alternative effective, clean and sustainable sources of energy. In the past two decades, researcher have been working on solar energy, because it is a clean and cost-free source of energy. The task is how to efficiently harvest the solar energy. Many research groups are working on finding new technologies and materials for harvesting solar energy more efficiently.

In recent years, low cost and high performance hybrid perovskites have attracted much attention from the scientific community.^1,2^ The flexible structural and compositional properties of hybrid perovskite materials make them preferable candidates for solar cell applications,^3,4^ light-emitting diodes,^5,6^ field-effect transistors,^7^ lasers, non-volatile memory devices^8–10^ and photodetectors,^11^*etc.* The use of hybrid halide perovskites with the structural formula ABX_3_, where A is an organic molecule like CH_3_NH_3_, NH_2_CH *etc.*, B is a cation such as Ge, Pb, Sn *etc.* and X represents anions such as Cl, Br, I, *etc.* has been explored. In 2009, hybrid halide perovskites as light absorbers were reported for the first time, with a power conversion efficiency (PCE) of 3.8%,^12^ which was useful to increase research in the photovoltaics field^13,18^ due to thhe tunable bandgap, charge carrier transport properties, and intense photoluminescence.^14–16,19–24^ Within a few years, the PCE of hybrid halide perovskites reached 22%, in comparison with traditional silicon-based solar cells.^14,25–28^

Despite good efficiency, three-dimensional hybrid perovskite shows low stability and device degradation when interacting with oxygen and moisture.^29–31^ The use of 2D forms of these structures is an efficient way to increase the stability, transport charge mobility, and flexibility.^32–35^ Therefore, it is important to study perovskite formation to understand the interaction between the inorganic PbI_3_ and organic CH_3_NH_3_ compound.

In experimental studies, Dou *et al.* were first to describe the single layer and multilayer thick single crystal of 2D (C_4_H_9_NH_3_)_2_PbBr_4_ perovskites, with a conformation of 2D perovskites separated by layers of PbBr_4_^−^. They showed the electronic properties of the 2D materials were different from the 3D bulk structure.^36^ As the absorber, 2D perovskites (BA)_2_PbI_4_ fabricated by Tsai *et al.* reached an efficiency of up to 12%, with greater stability and high efficiency.^37^ Also, the light–matter interactions, photoluminescence and photoresponse of 2D perovskites have been reported, and these 2D perovskites can be suitable for optoelectronic device applications.^38,39^ Experimentally Liu *et al.* have reported the 2D (MA)_2_PbI_4_, thin film as efficient in solar absorber applications and the electronic and optical properties.^40^ The halide perovskite’s structural and compositional properties modulation has been achieved in 0D, 1D, 2D, and 3D.^36^ It has been found that the 2D layered BA_2_GeI_4_ and BA_2_SnI_4_ are potential candidates for light-emitting devices.^41,42^ Also, Zhang *et al.*^43^ found that the bandgap, Rashba spin splitting, and effective mass vary with the thickness of 2D perovskites (Cs_2_PbI_4_, (MA)_2_PbI_4_) suggesting the 2D materials as good candidates for optoelectronic devices.

Up to now, many studies have been done on 2D hybrid perovskites. But how the effective transport and optical properties of the 2D hybrid perovskite CH_3_NH_3_PbI_3_ vary with thickness has rarely been studied. In this study, the transport charge mobility and optical absorption of multilayered CH_3_NH_3_PbI_3_ are systematically investigated at the density functional theory level. First, structural properties of monolayer and multilayer CH_3_NH_3_PbI_3_ are discussed. Then, with the electronic and charge carrier properties, the absorption properties of the multilayer hybrid are uncovered.

## 2 Methodology

Density functional theory (DFT) was employed with VASP implemented with the PAW method.^44–46^ The exchange–correlation functional (GGA-PBE) was used.^47,48^ It is well known that GGA-PBE cannot describe the weak interaction between CH_3_NH_3_ and PbI_3_ molecules. Therefore we applied Grimme DFT-D2,^48^ nonlocal density functional vdW (DFT-D2) implemented in VASP in the study. We also included SOC and HSE^49^ to get clear images of the electronic and transport properties of the 2D multilayer CH_3_NH_3_PbI_3_. In our previous work on the 2D single layer CH_3_NH_3_PbI_3_ perovskite, we have observed the underestimation and overestimation of the bandgaps using SOC and HSE calculations as shown in Fig S2 and S3.†^49,52^ The kinetic energy cut-off was set to 500 eV. To avoid successive interaction, a vacuum space of 20 Å was applied along the *Z*-direction. For the bilayer, trilayer and quadlayer, the distance between two successive layers was set as 3 Å, to check the interaction between layers and the effect of *N* layers on the cell phase. As we increase the number of layers, the *Z* direction increases up to ∼20 Å, ∼30 Å, and ∼45 Å for the bilayer, trilayer and quadlayer, respectively.

Structural optimization was performed using a (5 × 5 × 1) grid, and a (15 × 15 × 1) grid was used for the electronic properties. All atomic structures were optimized up to a force of 0.001 eV Å^−1^ with energy convergence about 10^−4^ eV for multilayer CH_3_NH_3_PbI_3_. The lattices agree well with experimental and theoretical studies.^50,53^

The formula for the perovskite crystal structure, with effective ionic radii for the ions at the A, B, and X sites, is shown in eqn (1).^54^1
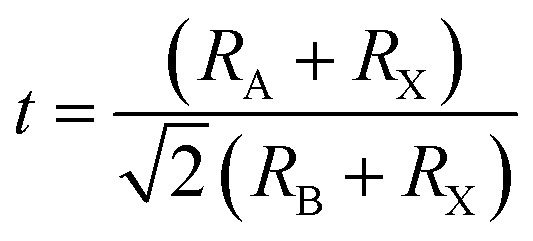


The dielectric function optical absorption coefficient can be calculated using the following equation.^53,55,58,59^2
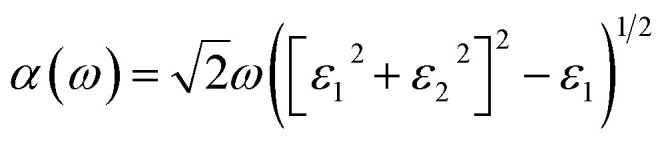
The transport properties of 2D multilayer CH_3_NH_3_PbI_3_ can be calculate using the Bardeen and Shockley^40,54^ method which was used in a previous report:^52^3
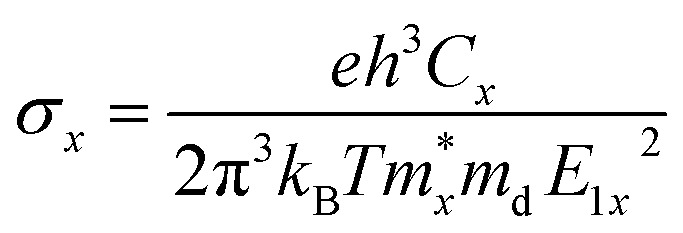


## 3 Results and discussion

### 3.1 Structural properties

**Fig. 1:**
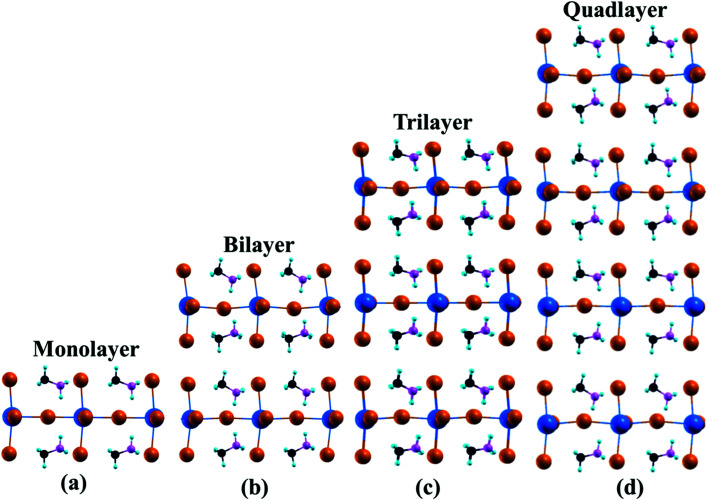
Atomic structures of multilayer hybrid halide perovskites. Pb, I, C, N, and H are represented in blue, orange, black, pink and sky blue, respectively.

The equilibrium configurations of multilayer CH_3_NH_3_PbI_3_ are shown in Fig. 1(a–d), while the structural parameters are listed in Table 1. The calculated in-plane lattice constants are *a* = 6.33 Å and *b* = 6.21 Å.^50–53,60^ The calculated bond length PbI is in the range of 3.09–3.27 Å for multilayer CH_3_NH_3_PbI_3_.

**Table 1:** Calculated lattice constants, bond lengths (*d*), and formation energy of 2D multilayer CH_3_NH_3_PbI_3_ structures

Structural parameter (Å)	*A*	*B*	*d*_(PbI_3_–CH_3_NH_3_)_	*d*_(C–C)_	*d*_(N–N)_	*d*_(Pb–Pb)_	*d*_(I–I)_	Formation energy (eV)
Monolayer	6.33	6.21	4.64	6.33	6.33	6.33	4.29	−70.8
Bilayer	6.34	6.21	4.50	4.12	5.37	10.23	3.91	−141.3
Trilayer	6.34	6.21	4.44	4.22	5.23	10.22	3.89	−211.9
Quadlayer	6.34	6.21	4.43	4.18	5.36	10.19	3.92	−282.4
Bulk	6.31, 6.33	6.31						

**Fig. 2:**
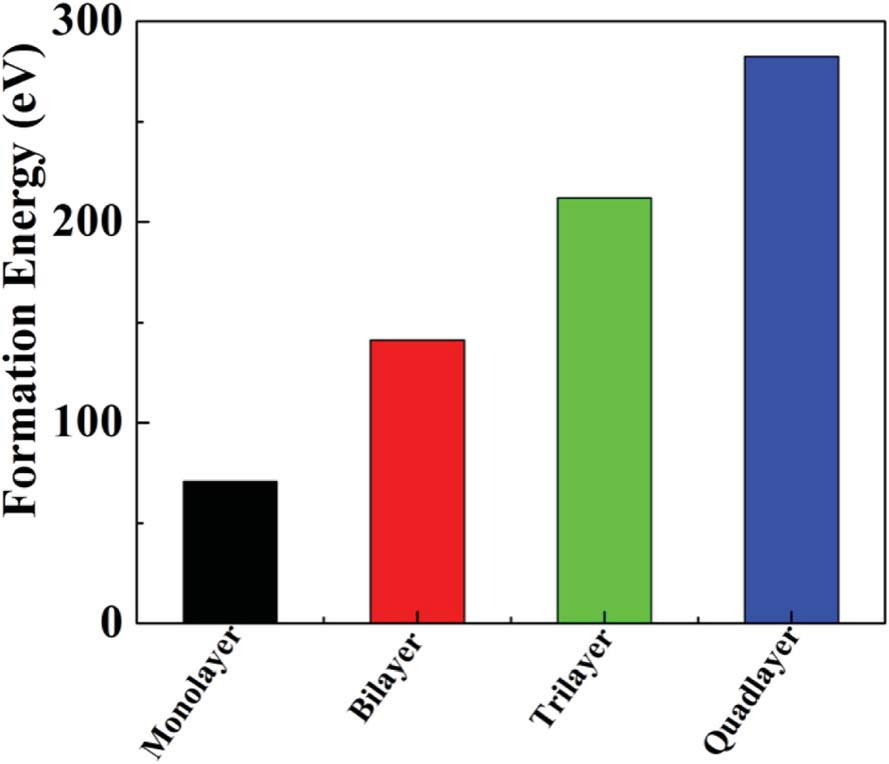
Formation energies of multilayer hybrid perovskites CH_3_NH_3_PbI_3_.

The calculated formation energy is [*E*_form_ = (*E*_layer_ − *nE*_Pb_ − *nE*_I_ − *nE*_CH_3_NH_3__)] where *E*_form_ is formation energy, *E*_layer_ is total energy of the layer, *E*_Pb_ is total energy of the Pb atoms, *E*_I_ is the total energy of the iodine atoms, *E*_CH_3_NH_3__ is the total energy of the CH_3_NH_3_ molecules. The formation energies of the multilayer perovskites are negative and increase with the corresponding increase in number of layers of CH_3_NH_3_PbI_3_, as shown in Fig. 2. The tolerance factor calculated using eqn (1) is 0.91 which is suitable for the hybrid halide perovskite structure.^38–40^

### 3.2 Electronic properties

**Fig. 3:**
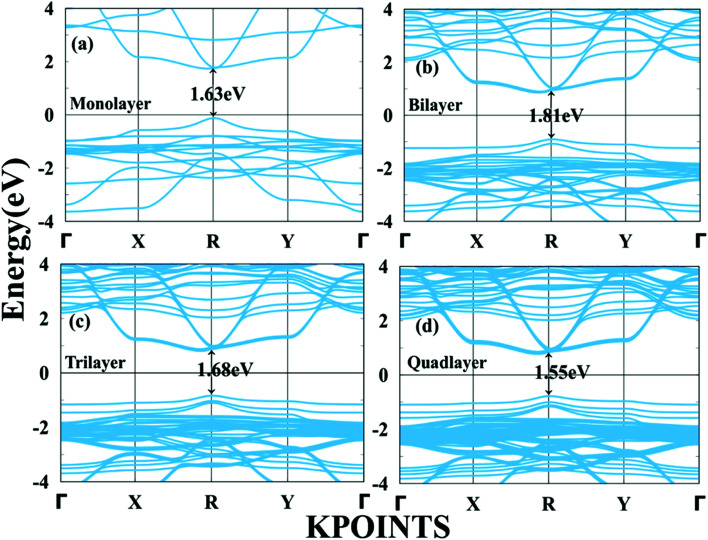
Calculated band structures of monolayer, bilayer, trilayer and quadlayer CH_3_NH_3_PbI_3_.

**Fig. 4:**
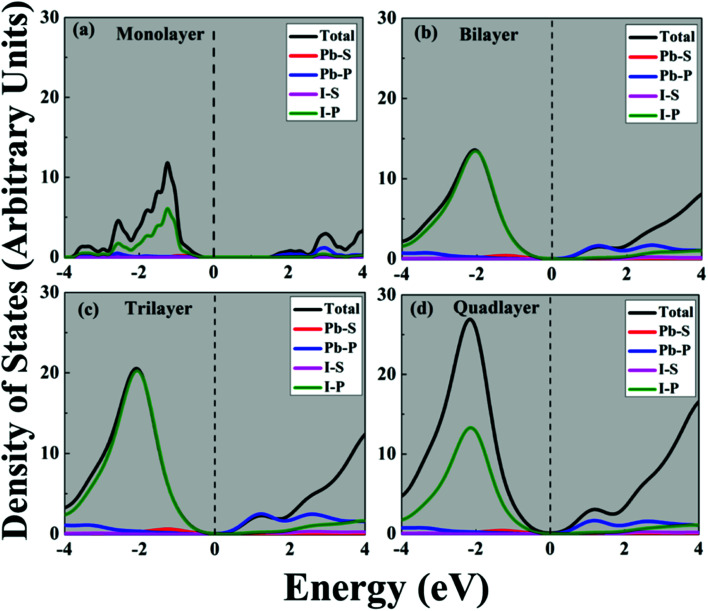
Calculated density of states of (a) mono-, (b) bi-, (c) tri- and (d) quadlayer CH_3_NH_3_PbI_3_.

The calculated band gap for the perovskite CH_3_NH_3_PbI_3_ monolayer is 1.63 eV; in the bilayer case it is 1.81 eV; for the trilayer 1.68 eV; and for the quadlayer, it is 1.55 eV as shown in Table 2 and Fig. 3. Similar to the bulk structure, multilayer CH_3_NH_3_PbI_3_ systems also have direct band gaps. The variation in the direct band gap occurred when we shifted from the monolayer to bilayer to trilayer to quadlayer due to an increase in localized states in the CBM and VBM near the Fermi level. Which appears as denser bands for the VBM compared to the CBM. In the case of the bilayer, the energy of the bottom of the near Fermi conduction band decreases and the energy of the top of the valence band increases, as shown in Fig. S5.† Which affects the band gap of the bilayer, which is higher than that of monolayer. Further we observed that when we shifted from bilayer to trilayer to quadlayer, the near Fermi CBM and VBM band energies decrease as we increase the numbers of layers, as shown in Fig. S5.† Further, we find that an indirect band gap occurs along the X to R point. The bandgap values are comparable with the corresponding bulk value of 1.50 eV.^61^ Moreover, Fig. 4 and Fig. S1† show the total density of states (TDOS) and that the edge of the valence band is mostly contributed by the Pb-6s and I-5p orbitals whereas the edge of the conduction band is dominated by the Pb-6p orbital.

**Table 2:** Calculated direct and indirect bandgaps of multilayer CH_3_NH_3_PbI_3_

	Bandgap PBE (eV)		Bandgap HSE (eV)	
	R–R	X–R	R–R	X–R
Monolayer	1.63	2.33	2.62	3.41
Bilayer	1.81	2.05	2.50	2.90
Trilayer	1.68	1.87		
Quadlayer	1.55	1.76		

### 3.3 Strain engineering

Further we have explored the strain dependent electronic properties of the multilayer CH_3_NH_3_PbI_3_ hybrid halide perovskites as shown in Table 3. Here we have applied both tensile and compressive strains up to 2%. We have observed that upon application of both types of strain the band gap of the system increases. For the monolayer it increases up to 2.01 eV for tensile strain, while in the cases of the bi-, tri- and quadlayer it increases up to 1.83 eV, 1.69 eV, 1.59 eV respectively. Near the Fermi level in the CBM most of the band line is occupied by non-antibonding states of the Pb-p and I-p orbitals and in the VBM most of the band lines are occupied by antibonding states of the Pb-s and I-p orbitals. As the number of layers increases from mono to bi to tri to quad, the corresponding band gap decreases as shown in Fig. S6, S7, S8 and S9† for the mono-, bi-, tri- and quadlayer, respectively. Similarly the variation in band gap occurs due to an increase in the localized state near the Fermi level. Fig. S4† shows the variation in formation energy and total energy with applied tensile and compressive strains for mono-, bi-, tri- and quadlayer CH_3_NH_3_PbI_3_. As we can observe, with tensile and compressive strain, the total energy of the system decreases with respect to the unstrained systems. From Fig. S4† we can observe the formation energy increase as we go from the monolayer to quadlayer.

**Table 3:** Calculated applied strain dependent bandgap of multilayer CH_3_NH_3_PbI_3_

Strain	Band gap (eV)				
	−2%	−1%	0%	+1%	+2%
Monolayer	2.01	1.98	1.63	1.98	1.97
Bilayer	1.78	1.82	1.81	1.83	1.83
Trilayer	1.65	1.66	1.68	1.69	1.67
Quadlayer	1.59	1.61	1.55	1.59	1.587

### 3.4 Carrier mobility

To evaluate the charge carrier efficiency in multilayer CH_3_NH_3_PbI_3_, we determined the carrier mobility *via* calculations of the deformation potential by applying compressive and tensile strain up to 2%. as displayed in Fig. S4† which shows formation energy under mechanical strain, and Fig. S10†shows the variation in CBM and VBM under both tensile and compressive strain. The directional dependent effective mass, as shown in Table 4, for the in-plane CBM electrons and VBM holes can be calculated using 
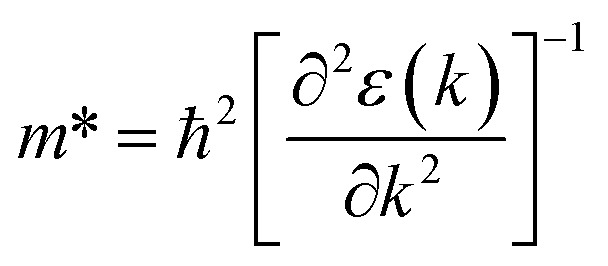
 where *ε*(*k*) is the wave vector *k*’s eigenvalue along the transport direction. This is an effective way to moderate transport properties. The charge carrier has some limitations in the bulk hybrid halide perovskite.

**Table 4:** Calculated effective mass (m*), deformation potential (*E*_1*x*_) and carrier mobility of multilayer CH_3_NH_3_PbI_3_

System	Electron				Hole			
	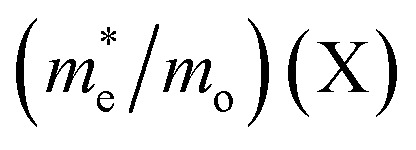	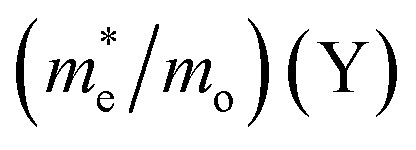	*E*_1*x*_ (eV)	*σ*_*x*_ (cm^2^ V^−1^ s^−1^)	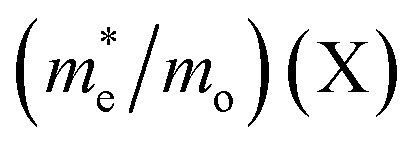	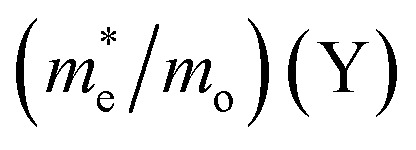	*E*_1*x*_ (eV)	*σ*_*x*_ (cm^2^ V^−1^ s^−1^)
Monolayer	3.89	3.92	7.57	414	1.09	1.51	4.49	1187
Bilayer	1.72	1.73	14.06	7.53	1.47	1.40	9.08	3.00
Trilayer	0.80	0.75	8.09	24.00	0.95	0.84	12.77	7.00
Quadlayer	0.74	0.72	10.5	21.08	0.87	0.79	11.97	11.50

Then, we compared the single layer (2D) transport properties^40,54^ of CH_3_NH_3_PbI_3_ with multilayer 2D CH_3_NH_3_PbI_3_ perovskite. In the comparison of the 2D single layer system with the 2D multilayer system, the average directional dependent effective mass of the electrons is decreased, from 3.92 to 0.72, while in the case of holes, the effective mass decreases from 1.51 to 0.79 as shown in Table 4. It is observed that the carrier mobility of the perovskite materials encompass a valuable range. The anisotropic nature of the carrier mobility is due to the difference in fabrication techniques and materials’ morphologies.^63^ 2D CH_3_NH_3_PbI_3_ has flexible structural properties, which affect the transformation from monolayer to bilayer and carrier mobilities are decreased. In the case of 2D multilayer hybrid halide perovskites, from bilayer to quadlayer, carrier mobilities increase from 3.00 cm^2^ V^−1^ s^−1^ to 24.00 cm^2^ V^−1^ s^−1^, which is consistent with previously reported 3D bulk halide perovskites, where 0.4 cm^2^ V^−1^ s^−1^ to 25 cm^2^ V^−1^ s^−1^ was found.^19,63^ We observed that as the number of layers increases from the monolayer to the quadlayer, the effective masses and carrier mobilities of the electrons and holes resemble the bulk nature of the CH_3_NH_3_PbI_3_ hybrid perovskites.^19,41,51,52,57,62,63^

### 3.5 Optical properties

**Fig. 5:**
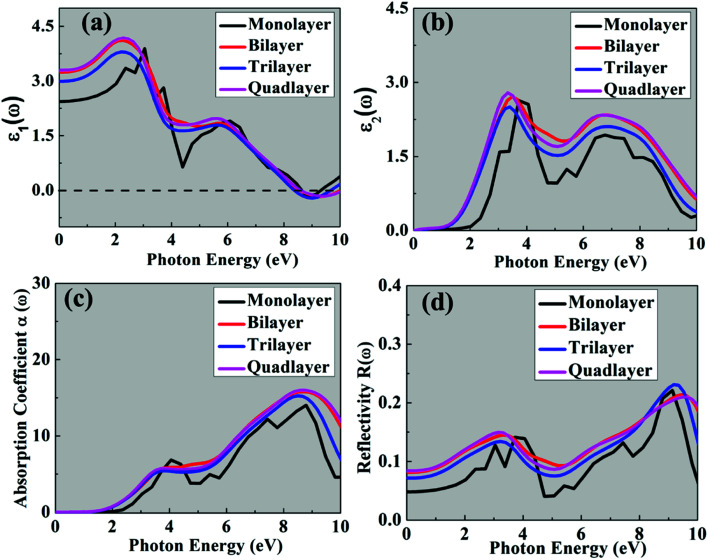
Optical properties of the 2D multilayer CH_3_NH_3_PbI_3_ perovskites: (a) *ε*_1_(*ω*), (b) *ε*_2_(*ω*), (c) absorption coefficient and (d) reflectivity.

The optical properties of multilayer CH_3_NH_3_PbI_3_ are shown in Fig. 5(a) and (b). We find that broadening of the peaks occurs with the increase in thickness for both *ε*_1_(*ω*) and *ε*_2_(*ω*). The calculated real *ε*_1_(*ω*) static dielectric constant of CH_3_NH_3_PbI_3_ increases from 2.43 to 3.40, monolayer to quadlayer, respectively. In the imaginary part of the dielectric function *ε*_2_(*ω*) the wide peak orientation is due to transition of Pb s to I p orbitals. Likewise, the absorption coefficient slightly increases from 14.1 × 10^5^ cm^−1^ for the monolayer to 16.0 × 10^5^ cm^−1^ for the quadlayer. Note that the calculated spectra lie in the range of 248 nm to 496 nm for multilayer CH_3_NH_3_PbI_3_. The calculated results are consistent with previously reported experimental results, containing CH_3_NH_3_PbI_3_ thin films with high PbI_2_ concentration.^8,17^ As well as the absorption coefficient is significantly more as compared with the bulk CH_3_NH_3_PbI_3_ (2.5 × 10^4^ cm^−1^ to 8.7 × 10^4^ cm^−1^).^8,52,56,58,59^ The high energy peaks at ∼4 eV occur due to the presence of a large number of interband transitions of the Pb s VBM to I p CBM at the R point.

In the optical reflectivity of the multilayer, shown in Fig. 5(d), the reflectivity spectra of multilayer CH_3_NH_3_PbI_3_ significantly enhance with an increase in photon energy. This reflects that the 1st peak is in the visible region and the 2^nd^ highest peak is in the ultraviolet region of the spectrum. We observed that the maximum light is in the UV region, compared with the visible region. Therefore, the light absorption by the CH_3_NH_3_PbI_3_ surface has been greatly enhanced in the visible (2 eV to 3.26 eV) as well as UV regions.

## 4 Conclusions

The calculated structural, electronic, and optical properties of the multilayer CH_3_NH_3_PbI_3_ hybrid halide perovskite system are investigated comprehensively with varying thickness using the DFT method. The formation energies, band gaps, and effective masses vary with the corresponding mono-, bi-, tri-, and quadlayer. We also observed that from mono- to quadlayer the bandgap decreases because of the transformation from 2D towards 3D. Due to strong antibonding states in the iodine and lead, high carrier mobilities are found with corresponding small effective masses. Besides, the multilayer has good optical properties with an absorption coefficient of about 10^5^ cm^−1^. The calculated absorption spectra lie between 248 nm and 496 nm, which leads to optical activity of the 2D multilayer CH_3_NH_3_PbI_3_ in the visible and ultraviolet regions. We believe that the reasonable absorption in the visible region with high carrier mobilities could make multilayer CH_3_NH_3_PbI_3_ a candidate material for efficient photovoltaic devices.

## Conflicts of interest

There are no conflicts to declare.

## Supplementary Material

RA-012-D2RA02874C-s001
