# Supplementary material for: Structural and compositional properties of 2D CH3NH3PbI3 hybrid halide perovskite: a DFT study
Source: RSC Adv. 2022 Sep 13;12(40):25924–31. doi: 10.1039/d2ra02874c (PMC9468801; doi:10.1039/d2ra02874c)
Supplement: RA-012-D2RA02874C-s001 [file RA-012-D2RA02874C-s001.pdf]

Electronic Supplementary Material (ESI) for RSC Advances.
This journal is © The Royal Society of Chemistry 2022

Supplementary Information

2D $CH_3NH_3PbI_3$ hybrid halide perovskite structural and compositional properties a DFT study

Sandip R. Kumavat[1], Geeta Sachdeva[2], Yogesh Sonvane[1,*],

Sanjeev K. Gupta[3]

[1]Advanced Materials Lab, Department of Physics, Sardar Vallabhbhai National Institute of Technology, Surat 395007, India

[2]Deparment of Physics, Michigan Technological University, Houghton, Michigan 49931, USA

[3]Computational Materials and Nanoscience Group, Department of Physics, St. Xavier's College,

Ahmedabad 38009, INDIA.

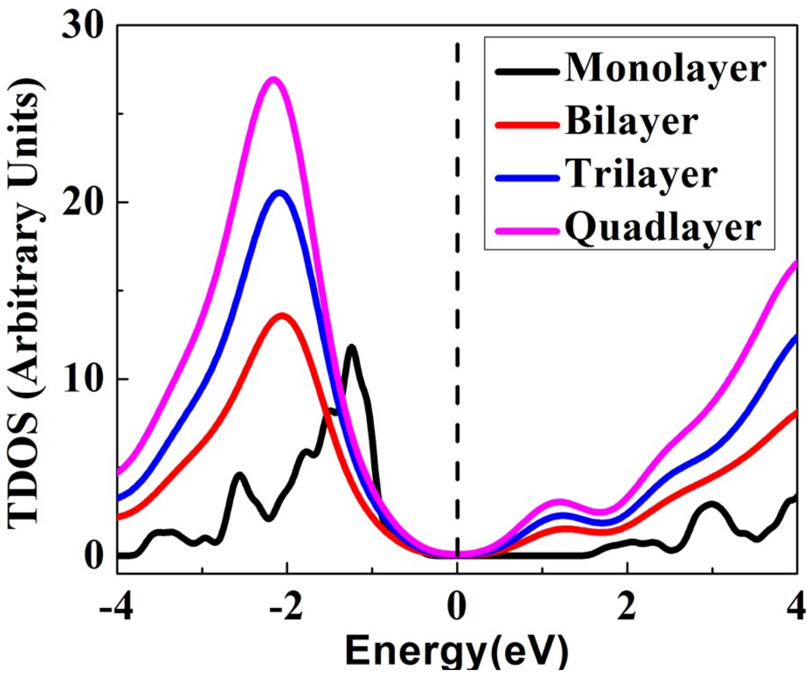

Figure S1: Calculated total density of states (TDOS) for mono, bi, tri, and quad layers of 2D $CH_3NH_3PbI_3$.

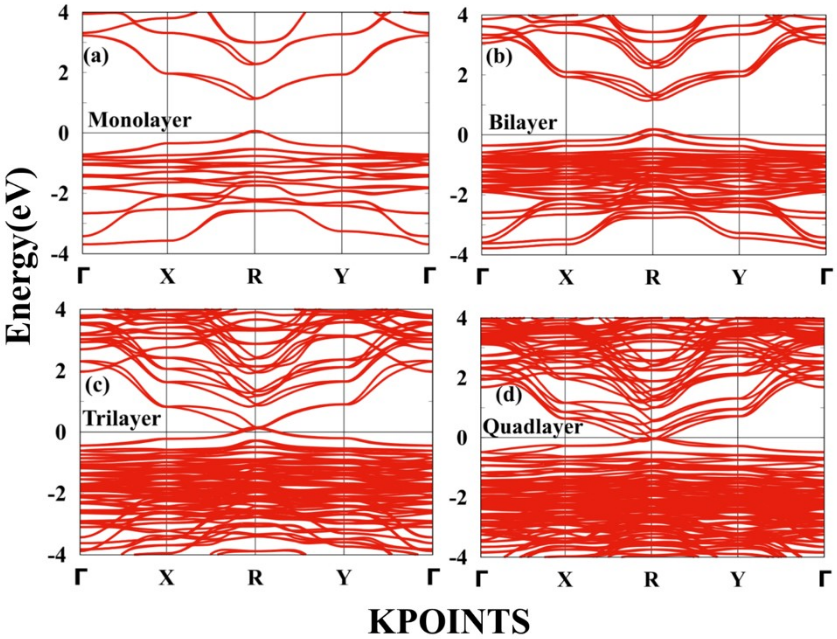

Figure S2. Calculated SOC band structure of (a)monolayer, (b)bilayer, (c)trilayer, and (d)quad-layer $CH_3NH_3PbI_3$.

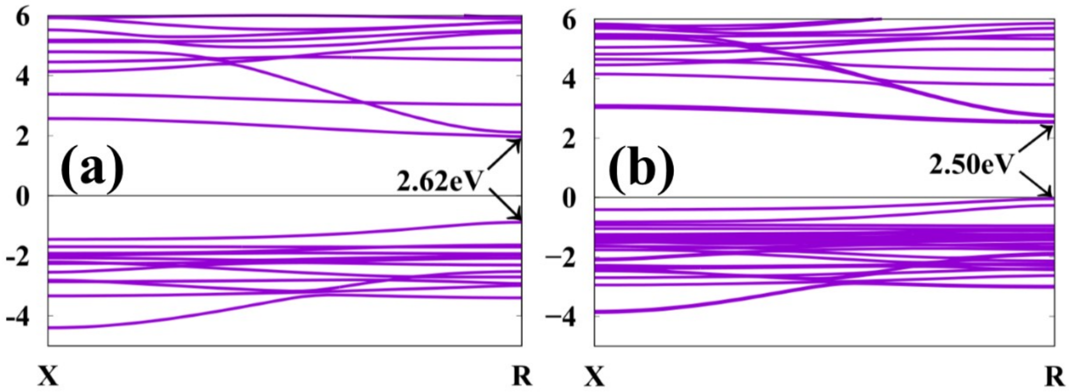

Figure S3. Calculated HSE band structure of (a)monolayer, (b)bilayer $CH_3NH_3PbI_3$.

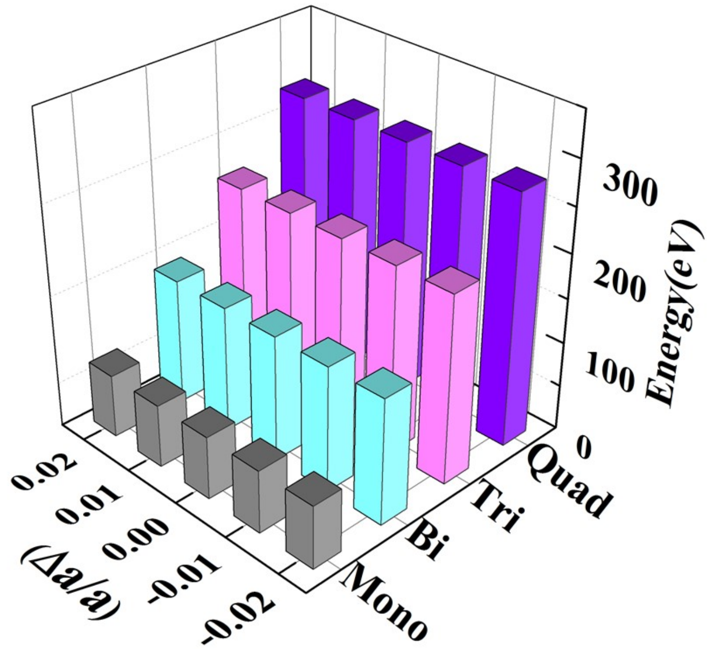

Figure S4. The variation of formation energy with applied tensile and compressive strain.

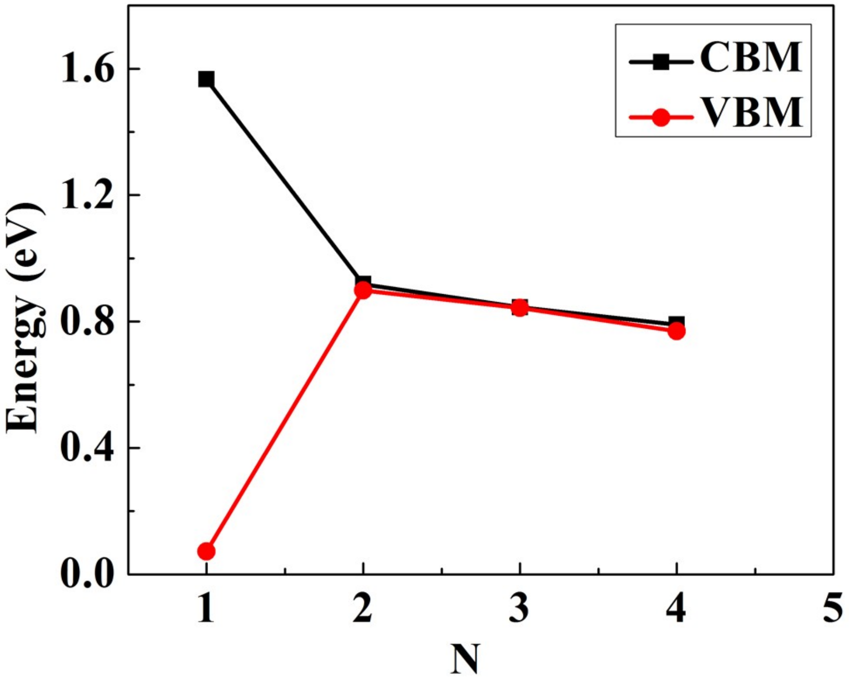

Figure S5. The variation in conduction band minimum (CBM) valence band maximum (VBM) Vs number of layers (N) of $CH_3NH_3PbI_3$.

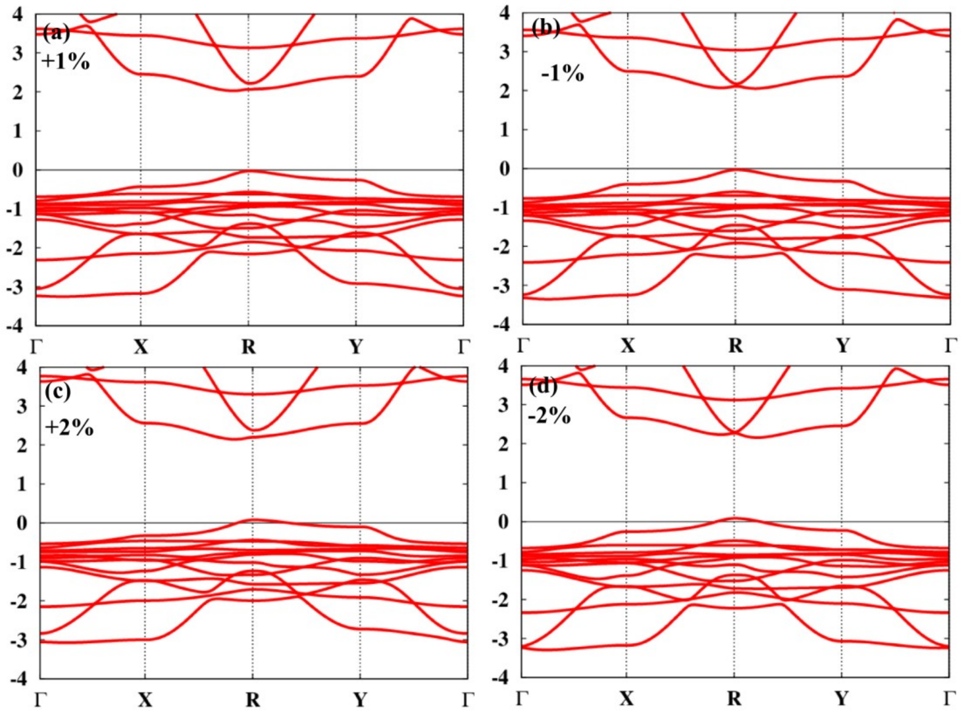

Figure S6. The variation in band gap under the tensile and compressive strain of monolayer of $CH_3NH_3PbI_3$.

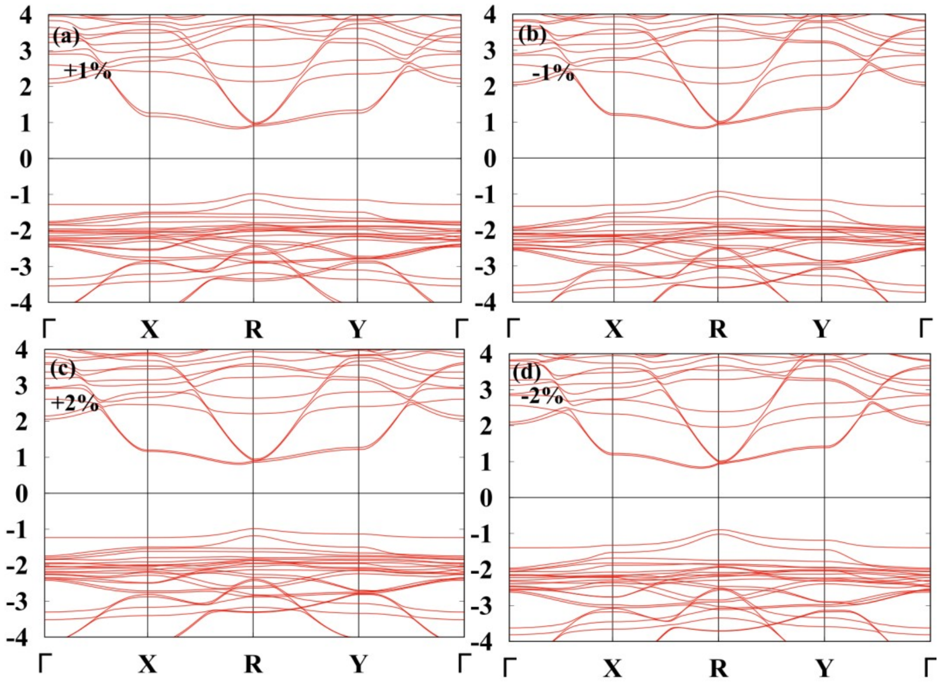

Figure S7. The variation in band gap under the tensile and compressive strain of bilayer of $CH_3NH_3PbI_3$.

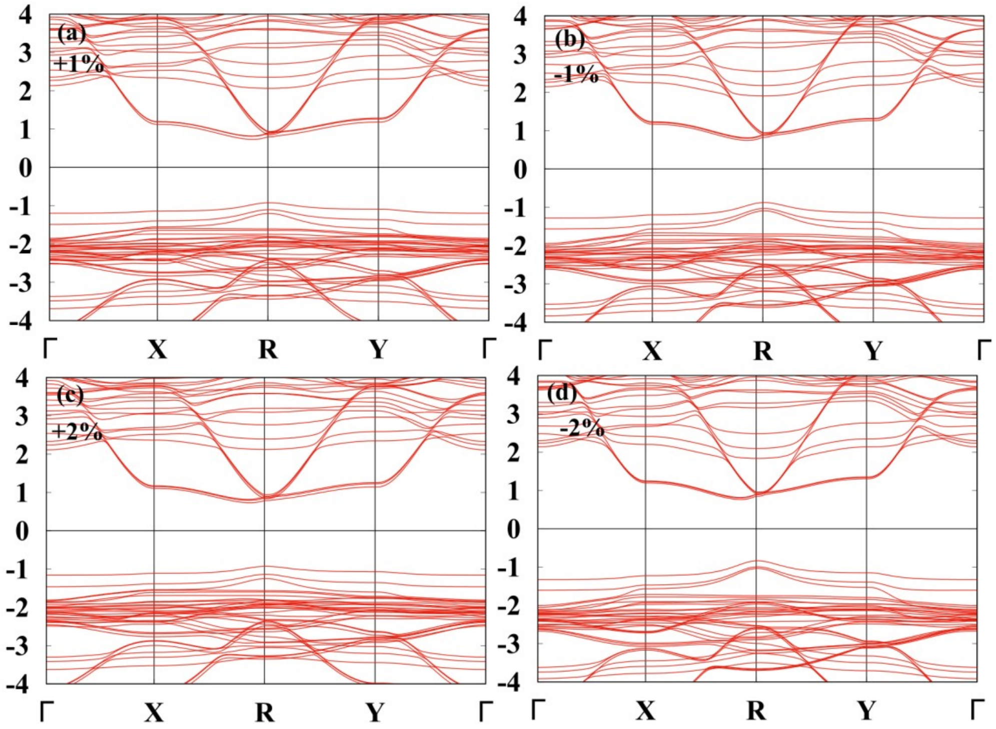

Figure S8. The variation in band gap under the tensile and compressive strain of trilayer of $CH_3NH_3PbI_3$.

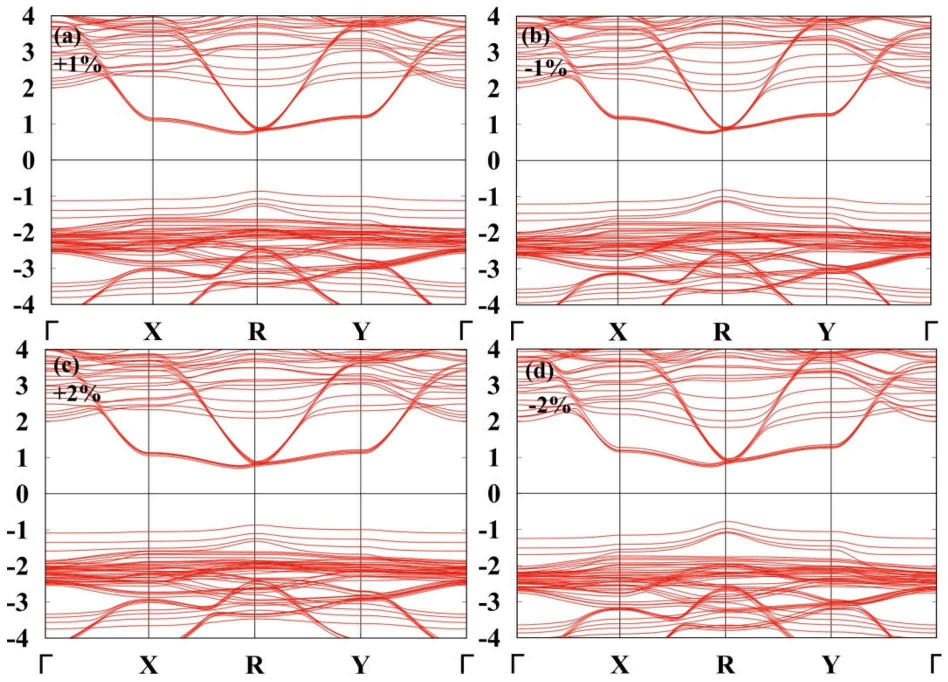

Figure S9. The variation in band gap under the tensile and compressive strain of quad-layer of $CH_3NH_3PbI_3$.

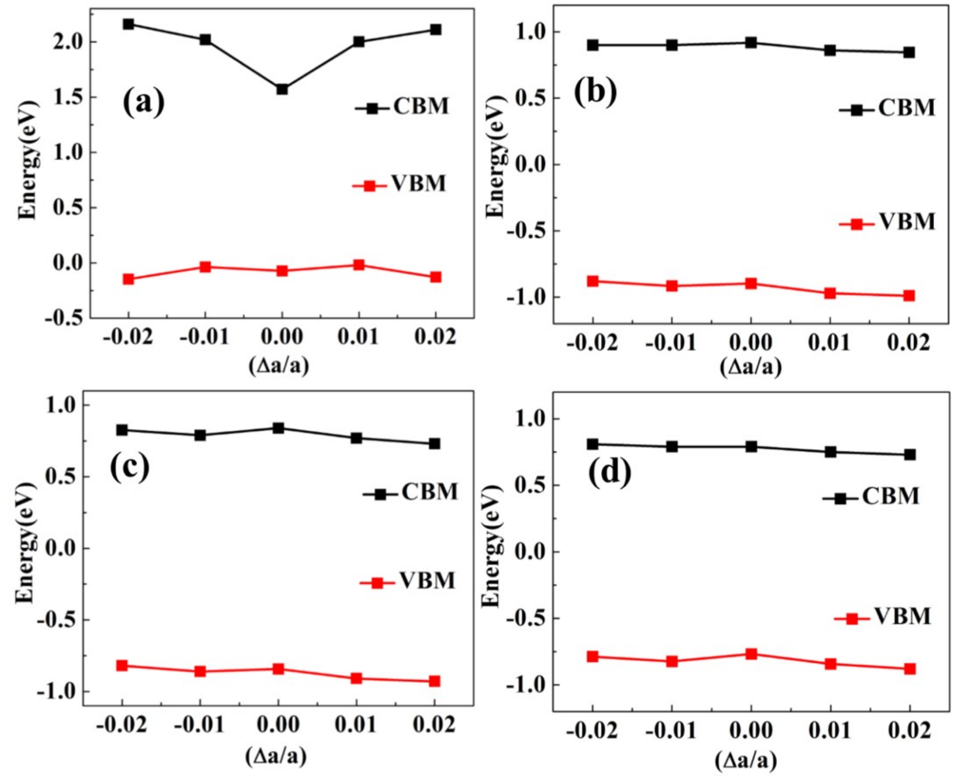

Figure S10. The variation in band gap under the tensile and compressive strain of (a)monolayer (b)bilayer (c)trilayer and (d) quad-layer of $CH_3NH_3PbI_3$.

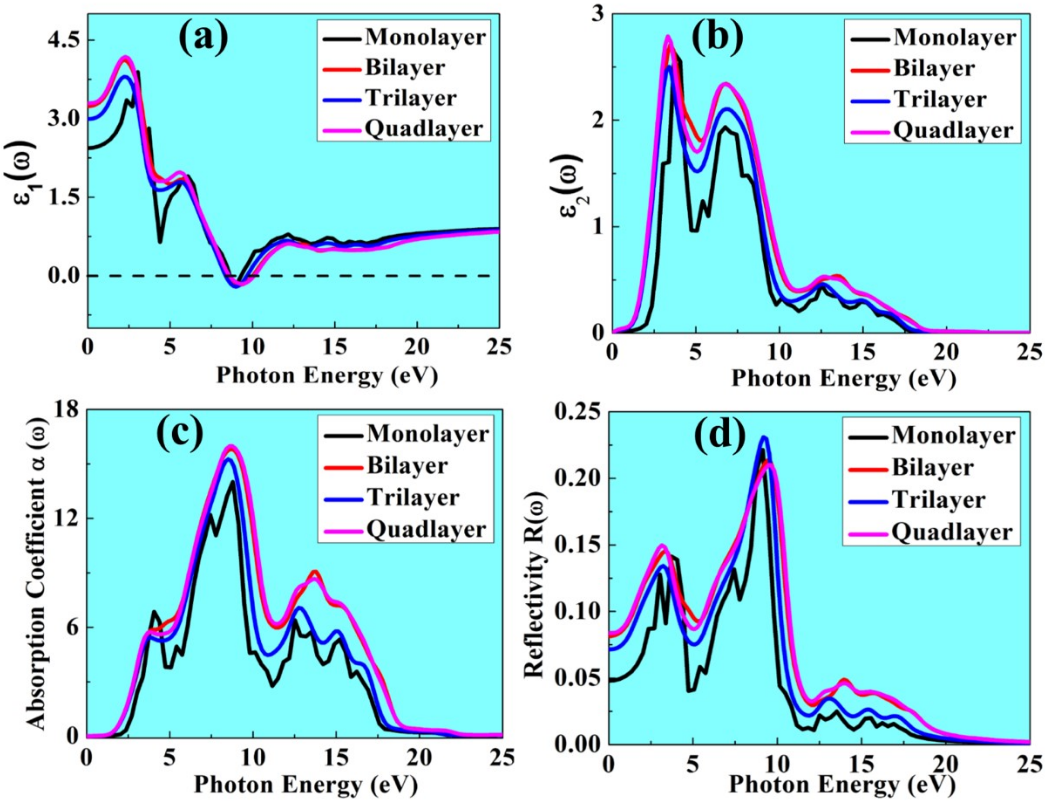

Figure S11. Optical properties of 2D multilayer $CH_3NH_3PbI_3$ perovskites with (a) real, (b) imaginary dielectric functions, (c) absorption coefficient and (d) reflectivity.
